# Infinite Selectivity of Wet SiO_2_ Etching in Respect to Al

**DOI:** 10.3390/mi11040365

**Published:** 2020-03-31

**Authors:** Imrich Gablech, Jan Brodský, Jan Pekárek, Pavel Neužil

**Affiliations:** 1 Central European Institute of Technology, Brno University of Technology, 612 00 Brno, Czech Republic; imrich.gablech@ceitec.vutbr.cz (I.G.); jan.brodsky@ceitec.vutbr.cz (J.B.); pekarek@vutbr.cz (J.P.); 2 Department of Microelectronics, Faculty of Electrical Engineering and Communication, Brno University of Technology, 616 00 Brno, Czech Republic; 3 Department of Microsystem Engineering, School of Mechanical Engineering, Northwestern Polytechnical University, Xi’an 710072, China

**Keywords:** SiO_2_ etching, microelectromechanical systems (MEMS), sacrificial layer, selectivity

## Abstract

We propose and demonstrate an unconventional method suitable for releasing microelectromechanical systems devices containing an Al layer by wet etching using SiO_2_ as a sacrificial layer. We used 48% HF solution in combination with 20% oleum to keep the HF solution water-free and thus to prevent attack of the Al layer, achieving an outstanding etch rate of thermally grown SiO_2_ of ≈1 µm·min^−1^. We also verified that this etching solution only minimally affected the Al layer, as the chip immersion for ≈9 min increased the Al layer sheet resistance by only ≈7.6%. The proposed etching method was performed in an ordinary fume hood in a polytetrafluorethylene beaker at elevated temperature of ≈70 °C using water bath on a hotplate. It allowed removal of the SiO_2_ sacrificial layer in the presence of Al without the necessity of handling highly toxic HF gas.

## 1. Introduction

Over the last 50 years, there has been a development in the semiconductor industry, primarily based on Si substrate for fabricating integrated circuits such as complementary metal oxide semiconductor (CMOS) devices [1]. Subsequently, due to its favorable published Young’s modulus value of 130 GPa for (100)-oriented Si [2], low thermal coefficient of expansion of 2.56 × 10^−6^ K^−1^, and high thermal conductivity of 157 W∙m^−1^∙K^−1^, Si has also been used to fabricate a large variety of microelectromechanical systems (MEMS) [3,4].

Many methods of Si micromachining, using Si as mechanical, thermal and electrical material, were developed during the evolution of MEMS technology, including wet anisotropic etching using either potassium hydroxide (KOH), tetramethyl ammonium hydroxide (TMAH) or ethylene diamine pyrocatechol solutions [5,6], and wet isotropic etching using a mixture of HF, HNO_3_ and CH_3_COOH, known as HNA solution [6]. Next, dry etching of Si was introduced, either anisotropic reactive ion etching (RIE) [7] and deep RIE (DRIE) [8], or semi-anisotropic etching using the plasma process [9], and finally isotropic etching using XeF_2_ vapor [10,11]. At the beginning, bulk micromachining prevailed [12,13] followed by more complex devices-based surface micromachining [14]. SiO_2_, typically in its low-stress form prepared by the plasma-enhanced chemical vapor deposition (PECVD) method, is another mechanical material commonly used in micromachining [15]. This material has full compatibility with Si processing and can also be used for high temperature deposition processes.

With the development of digital mirror device technology beginning in the 1980s [16], Al became another structural material used in MEMS fabrication. It can be used for numerous applications due to its compatibility with CMOS fabrication, such as making waveguides [17]. Al is also used as an electrical leadout for MEMS devices.

The etching of SiO_2_ by employing HF/NH_4_F solution (buffered oxide etch, BOE) can also be used, but unfortunately, it does etch Al; therefore, all structures made of Al have to be protected. Pinhole-free materials deposited by conventional technology such as PECVD do not exist; there are always some pinholes [18] allowing etch solution to penetrate through and to damage the Al layer underneath. The only option is protection by polymers as photoresist, but they cannot be applied for a long SiO_2_ release etch.

What about sacrificial etching of SiO_2_ in the presence of Al, though? Because, as is known, HF is only dissociated by H_2_O into H_3_O^+^ and F^−^ etches Al, the presence of water is therefore required. There have been several attempts to remove SiO_2_ using anhydrous gas HF [19] adding alcohol vapors using rather complex and expensive equipment achieving a slow etch rate of ≈15 nm∙min^−1^ of thermally grown SiO_2_ [20].

SiO_2_ in presence of Al has also been wet etched in liquid form using difficult-to-obtain 73% HF [21], achieving a high etch rate of 1.6 µm∙min^−1^ without attacking the Al layer, but this solution is classified as a weapon, as it can probably also be used for uranium enrichment, and thus the supply of this chemical is controlled.

A mixture of readily available 48% (28.9 M) HF/96% (18 M) H_2_SO_4_ solution, as well as only 48% HF solution, was also employed to conduct this SiO_2_ etching in the presence of Al [22], but there is a problem, as this etching produces water

SiO_2_ + 4HF → SiF_4_ + 2H_2_O (1)

at the device surface, causing dissociation of HF there and etching the Al layer.

There are numerous MEMS devices, such as inertial sensors, made of single-crystal silicon using silicon on insulator (SOI) substrates [23], inertial sensors integrated with CMOS based on α-Si [24], and bolometers made of α-Si [25] or α-SiGe [26], typically using SiO_2_ as sacrificial material employing anhydrous gas HF to remove the SiO_2_ layer [19]. This process works very well, but the capital investment is high, as HF is highly corrosive, as well as toxic, and the machine using HF gas has to be built to follow safety standards.

A similar problem arises with nitration of organic compounds to produce explosives with 99% HNO_3_. One of the reaction products is also H_2_O, which dilutes HNO_3_ and gradually stops the nitration process. The reaction is therefore conducted in the presence of highly hygroscopic H_2_SO_4_∙SO_3_ (oleum), binding H_2_O to itself, and keeping HNO_3_ concentrated, and thus active, for the nitration process [27].

In this contribution, we used a similar principle to etch SiO_2_ by 48% HF solution in the presence of oleum, concurrently binding H_2_O as an SiO_2_ etching product, keeping HF in non-dissociated form and thereby keeping the Al layer intact:

H_2_O + SO_3_ → H_2_SO_4_. (2)

Our method requires only minimal technical equipment, such as a fume hood and a hotplate and personal protective equipment such as glasses, face shield, chemically resistive gloves, and an apron resistive to strong acids.

## 2. Materials and Methods

### 2.1. Test Layout Design

**Figure 1:**
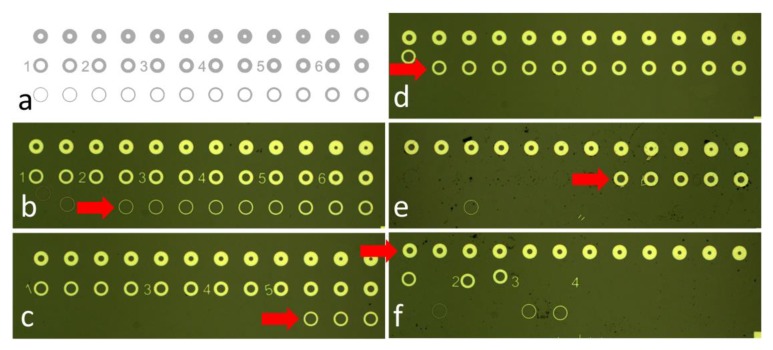
Test structure to determine the SiO_2_ etch via lateral under etching. (**a**) Structure layout containing structures with identical outer diameter and different width starting from 2 µm and ending with 20 µm with step of 0.5 µm. Photographs of structures after SiO_2_ etching for: (**b**) ≈1.5 min, (**c**) ≈3.0 min, (**d**) ≈4.5 min, (**e**) ≈6.0 min and (**f**) ≈7.5 min. The red arrow points at the torus shape with the smallest linewidth not fully undercut.

We designed a test pattern containing torus shapes with identical outer radius and variable inner forming a set of features with linewidth in a range from 2 µm to 20 µm with step of 0.5 µm a using Nanolithography toolbox [28]. The stepping of 0.5 µm gave us an etch rate resolution of 0.25 µm as the torus shapes were etched from both sides, which was sufficient for the purposes of this work (Figure 1a). Then, we fabricated a photolithography mask for contact printing using soda lime glass substrate with size of (≈127 × ≈127) mm^2^.

### 2.2. Sample Preparation

We used p-type Si (100) wafers with a diameter of ≈100 mm to fabricate the test structures. The wafers were oxidized to grow (398 ± 3)-nm-thick (mean ± standard deviation from three measurements using ellipsometry) SiO_2_. Then we deposited an Al layer using an e-beam evaporation technique with a thickness of ≈1.5 µm as measured by an in situ quartz crystal microbalance system. Subsequently, we coated the Al layer with a positive photoresist (PR) with a target thickness of ≈1.4 µm, and performed pre-exposure baking at ≈110 °C for ≈50 s on a hot plate in N_2_ atmosphere. We exposed the PR using an ultraviolet light source with a dose of ≈90 mJ∙cm^−2^ using a contact printer through a soda lime glass mask with a design as described above. Then we developed the PR using a TMAH-based developer for ≈60 s, washed it with deionized water and dried with an N_2_ flow.

Once we performed descumming process using O_2_ plasma for set duration, power and pressure of 60 s, 300 W and 7 Pa, respectively. The Al layer was subsequently etched by RIE using a mixture of Cl_2_ and BCl_3_ gas in the set ratio of 3:1. Then we removed the PR using 1-methyl-2-pyrrolidone, rinsed the wafer with propanol-2-ol (IPA), and dried it with a flow of N_2_. Finally, we cut the wafers into smaller pieces using the diamond scribing method into sizes of ≈(10 × 10) mm^2^, each containing a set of six test structures for etching evaluation.

### 2.3. Etch Solution Preparation

We mixed ≈50 mL of 48% HF (Sigma-Aldrich, Hampton, NH, USA) with ≈50 mL of 20% oleum (Fluke, St. Gallen, Switzerland) in a beaker made of polytetrafluorethylene (PTFE). The dilution heat warmed up the solution to elevated temperature up to its boiling point with white fumes coming out of the beaker; thus, a working in fume hood or a laminar box was essential as those fumes should consist of toxic HF and SO_3_ as well as non-toxic H_2_O. In the next step, we placed PTFE baker into bigger borosilicate glass beaker filled with water with its temperature set to ≈70 °C.

## 3. Experimental

We immersed six samples of devices as described above Al/SiO_2_ sandwich in the SiO_2_ etch solution for time in range from ≈1.5 min to ≈9 min with interval of ≈1.5 min to determine the etch rate. We also immersed two more samples into etch solution for ≈7.5 min, one with patterned Al and the other without. The first sample was used to determine the SiO_2_ etch rate via the undercutting of Al rings with different sizes. We also measured the Al thickness using a stylus type profilometer. The second sample was used to measure Al sheet resistance before and after its immersion. Each sample after etching was washed three times, twice with IPA followed by deionized H_2_O, then we dried it with a flow of N_2_.

## 4. Results and Discussion

**Figure 2:**
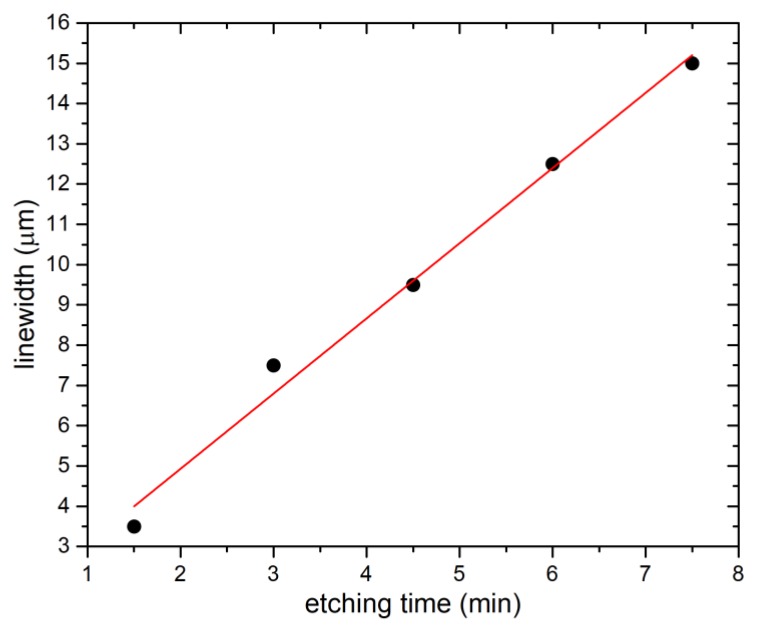
SiO_2_ etching as function of time. The curve was obtained as linear approximation for six etched samples for different time in range from ≈1.5 min to ≈7.5 min with an interval of ≈1.5 min.

First, we determined the etch rate of SiO_2_. We observed the etched structures using optical microscope and evaluated torus shapes washed away from the surface, i.e., completely undercut (Figure 1b–f). The last structures not fully undercut with their linewidths (Table 1) were 3.5 µm, 7.5 µm, 9.5 µm, 12.5 µm, and 15.0 µm for etching durations of ≈1.5 min, ≈3.0 min, ≈4.5 min, ≈6.0 min, and ≈7.5 min, respectively. The half of a linewidth of last surviving torus was plotted as a function of time (Figure 2), with its slope defining the SiO_2_ etch rate as (0.93 ± 0.05) µm∙min^−1^ (mean ± fitting error), which is ≈14× faster than BOE with 6:1 ratio of 48% HF and 40% NH_4_F [29]. 

**Figure 3:**
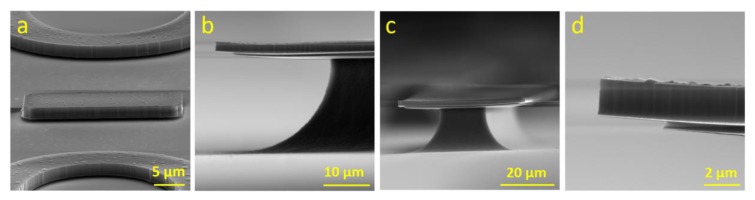
SiO_2_ etched with HF/oleum, showing the undercutting with Al layer intact: (**a**) Structures undercut by SiO2 etch solution leaving intact Al. (**b**) The same etching of SiO_2_ followed by partial Si removal by XeF_2_ vapor to enhance visibility of SiO_2_ etch boundary. (**c**) Different angle and magnification show whole structure (**d**) and corner detail.

Next, we etched samples in XeF_2_ vapor and set pressure and time to 0.33 Pa and 225 s, respectively, divided into 5 cycles to increase contrast between SiO_2_ and Al, and then checked them using scanning electron microscopy (SEM). SEM images show that the both Al and SiO_2_ layers were not mechanically damaged (Figure 3).

We measured the Al thickness by stylus profiler at the structures used for SiO_2_ etch rate testing and found that it was (1887 ± 46) nm (mean ± standard deviation from 3 measurements) of whole Al/SiO_2_ sandwich. These results show that the etching time had no influence on the sandwich thickness.

**Figure 4:**
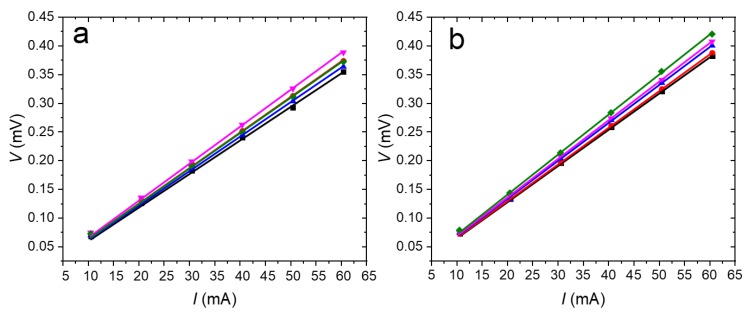
Graphs showing the value of *V* as a function of interrogating *I* from the four-point probe system at Al layer before (**a**) and after (**b**) dipping in SiO_2_ etch solution. The fitted slope *V*∙*I*^−1^ with an assumption of intercept of 0 V changed by ≈7.5%.

We measured Al sheet resistance (*R*_□_), before and after dipping it into SiO_2_ etch solution for ≈9 min. We used a custom-made four-point probe system, set the electric current (*I*) on the outer probes to the range from 10 mA to 60 mA, while monitoring the voltage (*V*) measured between the inner probes (Figure 4). Then we performed linear curve fitting determining the slope *V*∙*I*^−1^ and calculated the *R*_□_ value using the following equation:

(3) $$R_{□}=\frac{\pi }{ln2}\cdot \frac{V}{I}$$

We calculated the *R*_□_ value of the Al layer from the geometry factor constant and the slope (Figure 4) [30], before and after SiO_2_ etching for ≈9 min, as (27.8 ± 1.0) mΩ∙□^−1^ and (29.9 ± 1.2) mΩ∙□^−1^, respectively (both mean ± fitting errors from five measurements). The small increase in *R*_□_ values before and after SiO_2_ etching was probably caused by measuring on different area of substrate which can be influenced by Al thickness inhomogeneity and impurities on surface as the results from stylus profiler shown that the etch time had no influence on the Al thickness.

Here we summarize advantages and disadvantages of both, dry and wet etching systems. Dry etching is a convenient, user friendly and safe technique using a load lock system practically eliminating an option of an operator to get into contact with the HF gas. Also, there is no need for critical dry release of the structure, as there is no liquid involved in the process. The disadvantage is often prohibited cost of the system as well as its slow etch rate of ≈15 nm∙min^−1^.

The wet etch proposed in this contribution has a high etch rate of (0.93 ± 0.05) µm∙min^−1^ (mean ± fitting error), as well as requiring practically no special equipment besides a fume hood, PTFE beaker and personal protective equipment. The disadvantage is requirement of critical dry release or similar method to prevent the MEMS structures to collapse. Also, this etching technique should only be performed by skilled personnel, as they will be dealing with hazardous chemicals. Disposing the etch solution should be done in an appropriate manner using conventional HF types of waste.

## 5. Conclusions

We proposed and verified the wet SiO_2_ etching method with excellent selectivity towards Al, practically leaving the Al layer intact. HF solution in the absence of H_2_O does not etch Al; thus, we used 48% HF in combination with oleum to etch SiO_2_ by HF, with concurrent removal of H_2_O, product of SiO_2_ etching by hygroscopic oleum. We tested this idea by etching thermally grown SiO_2_, achieving a very high etch rate of (0.93 ± 0.05) µm∙min^−1^ (mean ± fitting error from three measurements), ≈14× faster in comparison with a typical 6:1 BOE etch rate ≈70 nm∙min^−1^. During this process, the SiO_2_ and the Al layer present at the tested chip remained intact, as the sheet resistance before and after exposure to the solution stayed almost the same. The presented method is a simple alternative to anhydrous gas HF etching of SiO_2_, sacrificial etching, with the Al layer presented on the substrate conducted by complex gas systems.

## Figures and Tables

**Table 1:** Line width of the layout shown in Figure 1a having three rows and 12 columns. Please note that there was a mistake on the layout, and thus the linewidths with size of 6.5 µm and 7.0 µm are missing.

Column/Row	1	2	3	4	5	6	7	8	9	10	11	12
1	15.0	15.5	16.0	16.5	17.0	17.5	18.0	18.5	19.0	19.5	20.0	20.0
2	9.0	9.5	10.0	10.5	11.0	11.5	12.0	12.5	13.0	13.5	14.0	14.5
3	2.0	2.5	3.0	3.5	4.0	4.5	5.0	5.5	6.0	7.5	8.0	8.5
